# List of Accidents, Discharged from the Pennsylvania Hospital, from Sept. 19, to Oct. 18, 1838

**Published:** 1838-10-24

**Authors:** J. F. Meigs

**Affiliations:** Resident Surgeon


### CLINICAL REPORTS.

### PENNSYLVANIA HOSPITAL.

*List of Accidents, discharged from the Pennsylvania Hospital, from Sept.* 19, *to Oct.* 18, 1838.

[Reported by J. F. Meigs, M. D., Resident Surgeon.]

Discharged, cured, October 5th, a case of simple fracture of both bones of the leg, having been treated for the first forty-two days with the fracture-box, afterwards with paste-board splints, which were continued until a few days before the discharge, which was on the seventy-first day after the accident.

Discharged, cured, on the 3d of October, a case of simple fracture of both bones of the leg, which had been treated for the first forty-four days with the fracture-box, then with paste-board splints, for the space of two weeks, and discharged on the eighty-ninth day after the accident. The case of fractured humerus, within the anatomical neck, reported in No. 20, was discharged, cured, with perfect use of the limb on the 13th of October, the splints having been removed on the thirty-seventh day, and a sling merely substituted, the patient leaving the house on the fortieth day after the accident.

Discharged, cured, on the 3d of October, the forty-fourth day after the accident, a case of fractured fibula, which had been treated for the first thirty-nine days with the fracture-box, afterwards with the paste-board splints.

Discharged, cured, October 13th, the forty-first day after the accident, a case of simple fracture of the fibula above its middle, in a boy eleven years old, which had been treated for the first twentysix days with the fracture-box, afterwards with the paste-board splints.—Admitted, September 24th, a case of lacerated wound of the integuments over the right mastoid process, caused by a blow from a stone; there was slight hæmorrhage from the ear, no fracture of the cranium, nor any symptoms of concussion or compression of the brain. Wound dressed with lint and adhesive strips; symptoms of mania-a-potu came on, which were relieved by opiates and a blister to the back of the neck, the patient being discharged cured, in eleven days.—Admitted, September 30th, a case of strangulated inguinal hernia of the right side. The hernia in this case was of long standing, the patient having worn a truss for years; on the morning of the day of his admittance, he had removed the truss for a short time, during which the bowel descended, and he found it impossible to reduce it; he soon afterwards was attacked with severe pain and constriction in the abdomen, upon which he sent for a physician, who bled him largely and attempted the reduction of the bowel; but not succeeding he was brought to the hospital, about six hours after the accident, when he complained of severe pain and constriction in the abdomen, and of soreness in the tumour. He was immediately placed in a warm bath, and as he had been costive for several days, an injection was ordered, which produced a free stool. He remained in the bath for the space of half an hour, during which time the truss was used without effect, excepting slightly to diminish the size of the tumour, which appeared to consist entirely of intestine filled with hardened fæces. He was now placed in bed, and the taxis again used without effect, upon which, as the symptoms were not very urgent, cloths, wrung out with water as hot as he could bear, were applied to the scrotum for a short time, and the taxis being again attempted, the reduction was effected about two hours and a half after his entrance. He was ordered to keep quiet, and as no bad symptoms supervened, was discharged in a few days.

Admitted, October 13th, a case of gun-shot wound of the abdomen, the patient having shot himself a few hours previously with a pistol, loaded with ball. When admitted, he was very much exhausted, the face being pale, the extremities cool, and the pulse frequent and weak with vomiting of small quantities of blood. Upon examination it was found that the ball had entered opposite the cartilage of the eighth rib of the left side; the hæmorrhage was very slight, and the ball could not be found; and lint was applied over the wound by means of adhesive strips; the patient placed in bed, and entire rest enjoined. Died thirty hours after admittance. Upon examination, it was found that the ball had passed through the cartilage of the rib, at the point where it joins the osseous portion, through the diaphragm, had perforated the left edge of the liver, passed through both the anterior and posterior fæces of the cardiac extremities of the stomach, struck the kidney, causing a considerable ecchymosis beneath its external coat, and then passed out of the cavity of the abdomen, being removed from just beneath the integument of the loins.—Admitted, October 8th, a case of severe contusion of the back caused by a fall. When admitted, several days after the accident, there was considerable swelling and contusion over the dorsal vertebræ, with severe pain moving; there was no paralysis of any part, the functions of the rectum and bladder being perfect. The man was placed in bed, cupped repeatedly in the back, and ordered low diet, under which treatment he recovered, and left the house well at the end of nine days.

